# Machine learning-based prediction of survival prognosis in cervical cancer

**DOI:** 10.1186/s12859-021-04261-x

**Published:** 2021-06-16

**Authors:** Dongyan Ding, Tingyuan Lang, Dongling Zou, Jiawei Tan, Jia Chen, Lei Zhou, Dong Wang, Rong Li, Yunzhe Li, Jingshu Liu, Cui Ma, Qi Zhou

**Affiliations:** 1 Key Laboratory of Biorheological Science and Technology (Chongqing University), Ministry of Education, Bioengineering College, Chongqing University, Chongqing, 400044 People’s Republic of China; 2 Department of Gynecologic Oncology, School of Medicine, Chongqing University Cancer Hospital, , Chongqing University, Chongqing, 400030 People’s Republic of China; 3 Chongqing Key Laboratory of Translational Research for Cancer Metastasis and Individualized Treatment, School of Medicine, Chongqing University Cancer Hospital, Chongqing University, Chongqing, 400030 People’s Republic of China; 4 School of Mathematics and Statistics, Changchun University of Technology, Changchun, 130012 People’s Republic of China; 5 Singapore Eye Research Institute, The academia, 20 College Road, Discovery Tower Level 6, Singapore, 169856 Singapore; 6 Department of Ophthalmology, Yong Loo Lin School of Medicine, National University of Singapore, Singapore, Singapore; 7 Duke-NUS Medical School, Ophthalmology and Visual Sciences Academic Clinical Research Program, National University of Singapore, Singapore, Singapore; 8 Department of Pediatric Hematology, First Hospital of Jilin University, Changchun, 130023 Jilin People’s Republic of China

**Keywords:** Cervical cancer, miRNAs, Machine learning, Survival prediction, Support-vector machines

## Abstract

**Background:**

Accurately forecasting the prognosis could improve cervical cancer management, however, the currently used clinical features are difficult to provide enough information. The aim of this study is to improve forecasting capability by developing a miRNAs-based machine learning survival prediction model.

**Results:**

The expression characteristics of miRNAs were chosen as features for model development. The cervical cancer miRNA expression data was obtained from The Cancer Genome Atlas database. Preprocessing, including unquantified data removal, missing value imputation, samples normalization, log transformation, and feature scaling, was performed. In total, 42 survival-related miRNAs were identified by Cox Proportional-Hazards analysis. The patients were optimally clustered into four groups with three different 5-years survival outcome (≥ 90%, ≈ 65%, ≤ 40%) by K-means clustering algorithm base on top 10 survival-related miRNAs. According to the K-means clustering result, a prediction model with high performance was established. The pathways analysis indicated that the miRNAs used play roles involved in the regulation of cancer stem cells.

**Conclusion:**

A miRNAs-based machine learning cervical cancer survival prediction model was developed that robustly stratifies cervical cancer patients into high survival rate (5-years survival rate ≥ 90%), moderate survival rate (5-years survival rate ≈ 65%), and low survival rate (5-years survival rate ≤ 40%).

**Supplementary Information:**

The online version contains supplementary material available at 10.1186/s12859-021-04261-x.

## Background

Cervical cancer is the main cause of women deaths worldwide which accounts for more than 520,000 new cases and 260,000 deaths each year [1, 2]. Although vaccines against the prime carcinogenic human papilloma virus (HPV) types are available commercially, the proportion of women receiving the vaccine is still low, especially in developing countries [3, 4]. Furthermore, despite effective treatment of early cervical cancer with surgery and radiation therapy, late cervical cancer is usually uncontrollable [5, 6].

Survival prediction after first diagnosis is important for both disease specialist and patients or their family members. First, as the survival ability of the cancer patients largely depends on the malignancy of the cancer cells, accurately forecasting the prognosis would be helpful for estimating the degree of malignancy and the time point of disease progression [7, 8]. On the other hand, patients and the families can set appropriate goals base on the accurate survival prediction. As the result, the timely prevention and treatment would be made and the worse treatment decision, such as over-treatment or late palliative care, would be effectively avoided.

**Fig. 1:**
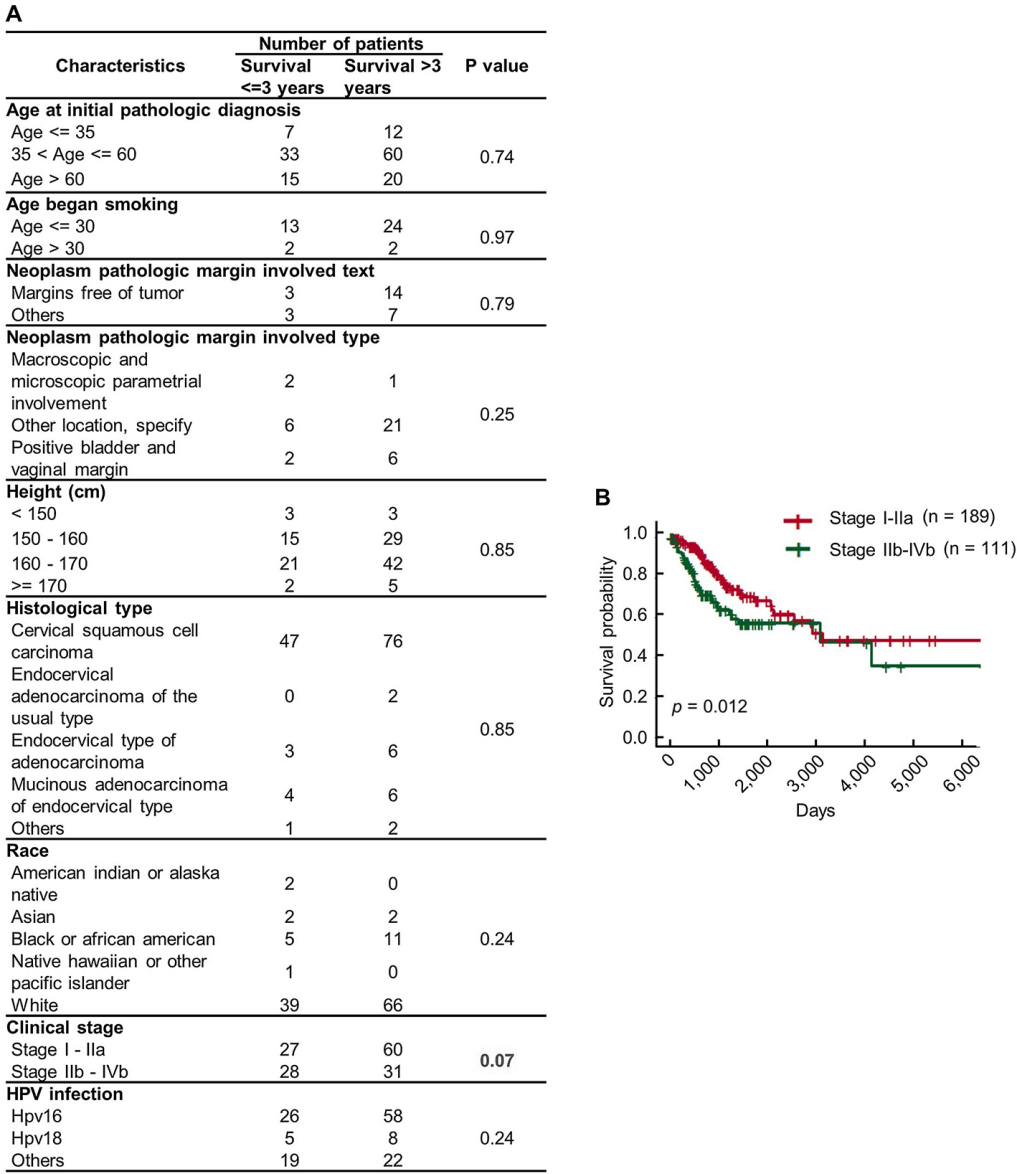
The relationship between clinical features and survival of cervical cancer. **a** Chi-square analysis of the association between clinical features and 3-years survival of cervical cancer patients. **b** Kaplan–meier analysis of the association between clinical stage and the survival of cervical cancer. Chi-square was performed using chisq.test() function in “stats” (version 3.6.2) package. Kaplan–meier analysis was performed using Surv() and survfit() function in “survival” (version 3.2–10) package. The Kaplan–meier plot was performed using ggsurvplot() function in “survminer” (version 0.4.9). RStudio (version 3.6.1, RStudio, Inc.) is used

However, accurate prediction of survival of cervical cancer patients is still challenging due to the heterogeneity of the cancer cells. In general, the cervical cancer patients were stratified into different groups base on cancer staging systems, TNM classification [9–11], for example. However, the molecular features have been rarely considered in such staging systems that numbers of subtypes of patients with different survival outcomes would be existed in one specific TNM stage. Furthermore, clinical features, such as TNM stage, could not provide enough information for survival prediction. For example, we investigated the correlation between several clinical features (including age at initial pathologic diagnosis, age began smoking, neoplasm pathologic margin involved text, neoplasm pathologic margin involved type, height, histological type, race, clinical stage, HPV infection) and the survival of cervical cancer patients included in The Cancer Genome Atlas (TCGA) dataset and found that only clinical stage is relevant, however, its discrimination ability is still not enough for accurate prediction (Log-rank *p* = 0.012) (Fig. 1a and b). Thus, novel prediction strategies were urgently needed to be developed.

Molecular features (such as gene or noncoding RNA expression levels, gene mutation, copy number variation, etc.) imply substantial information about cancer cells, including malignant level, metastasis ability and therapeutic sensitivity, etc. [12]. Several cancers (colorectal, breast, and cervical cancers, for example) have been stratified into subtypes base on the molecular profiles provided by rapidly developing database of cancer molecular information, such as TCGA [13–15]. Thus, development of molecular features-based prediction model keeps the promise for improving the accuracy of cancer survival prediction model. Furthermore, as a part of artificial intelligence, machine learning (ML) provides a solution for accuracy improvement of cancer survival prediction model. Machine learning is a process for analysis of big data, that was characterized as learning form mistakes and experiences [16, 17]. Several machine learning models, such as support vector machines (SVM), have been widely used for development of prediction model base on electronic medical record, images as well as molecular features of cancer cells [18–22].

Thus, the objective of this study is to develop a novel molecular features-based machine learning cervical cancer survival prediction model (CCSPM) with high performance. MiRNAs were chosen as features and Cox-PH, K-means clustering and SVM algorithms were used for survival-related features identification, features-based objectives stratification, and prediction model development, respectively. The results of this study would improve the forecasting capacity of CCSPM and be helpful for cervical cancer management.

## Results

### Insufficient discriminative ability of clinical features for development of cervical cancer survival prediction model

Numbers of features with high discriminative ability is essential for development of a prediction model [23]. To develop the survival prediction model for cervical cancer, we investigated whether clinical features possess high discriminative ability for stratifying cervical cancer patients with different survival outcome. The information of clinical features and the survival of cervical cancer patients was downloaded from TCGA database [15]. The summary of the clinical information was given in Table 1. The cervical cancer patients were stratified base on 3-years survival outcome and the clinical features were grouped as shown in Fig. 1a. Chi-square analysis was performed to determine whether clinical features were correlated with 3-years survival outcome. Unfortunately, no clinical features were found to be correlated with 3-years survival of cervical cancer base on Chi-square analysis (Fig. 1a), while, clinical stage has shown its potential (*p* = 0.07). We next performed Kaplan–meier analysis to confirm the link of clinical stage with cervical cancer survival. As shown in Fig. 1b, although the two groups of patients stratified by clinical stage exhibited statistically different survival outcome, its discriminative ability is still not enough for development of a survival prediction model (Log-rank *p* value = 0.012). These results suggested that clinical features, including clinical stage, could not provide enough information for development of CCSPM.

**Table 1:** Summary of the clinical information of cervical cancer patients included in TCGA database

Characteristics	Cohort (n=307)	
	No.	%
*Age at initial pathologic diagnosis*		
Age <= 35	56	18.24
35 < Age <= 60	192	62.54
Age > 60	59	19.22
NA	0	0.00
*Age began smoking*		
Age <= 30	75	24.43
Age > 30	10	3.26
NA	222	72.31
*Neoplasm pathologic margin involved text*		
Margins free of tumor	23	7.49
Others	17	5.54
NA	267	86.97
*Neoplasm pathologic margin involved type*		
Macroscopic and microscopic parametrial involvement	10	3.26
Other location, specify	40	13.03
Positive bladder and vaginal margin	13	4.23
NA	244	79.48
*Survival (years)*		
<= 3	55	17.92
> 3	92	29.97
Loss to follow up < 3	160	52.12
*Height (cm)*		
< 150	15	4.89
150 - 160	119	38.76
160 - 170	111	36.16
>= 170	19	6.19
NA	43	14.01
*Histological type*		
Adenosquamous	6	1.95
Cervical squamous cell carcinoma	254	82.74
Endocervical adenocarcinoma of the usual type	6	1.95
Endocervical type of adenocarcinoma	21	6.84
Endometrioid adenocarcinoma of endocervix	3	0.98
Mucinous adenocarcinoma of endocervical type	17	5.54
NA	0	0.00
*Race*		
American indian or alaska native	8	2.61
Asian	20	6.51
Black or african american	30	9.77
Native hawaiian or other pacific islander	2	0.65
White	211	68.73
NA	36	11.73
*Clinical stage*		
Stage I - IIa	189	61.56
Stage IIb - IVb	111	36.16
NA	7	2.28
*HPV infection*		
Hpv16	172	56.03
Hpv18	39	12.70
Others	73	23.78
NA	23	7.49

NA: Not available, HPV: human paplillomavirus

### Data preprocessing of TCGA miRNAs expression data

We next investigate whether the expression of microRNAs (miRNAs) could be served as features for development of CCSPM. The miRNA expression data from TCGA database was used in this study [15]. The data preprocessing was first performed; data preprocessing plays important roles for statistical analysis of big data, including elimination of the impact from unquantified samples and features, missing values and outliers, reduction of batch-effects and experimental deviation, and normalization of the range of independent features, etc. [24].

**Fig. 2:**
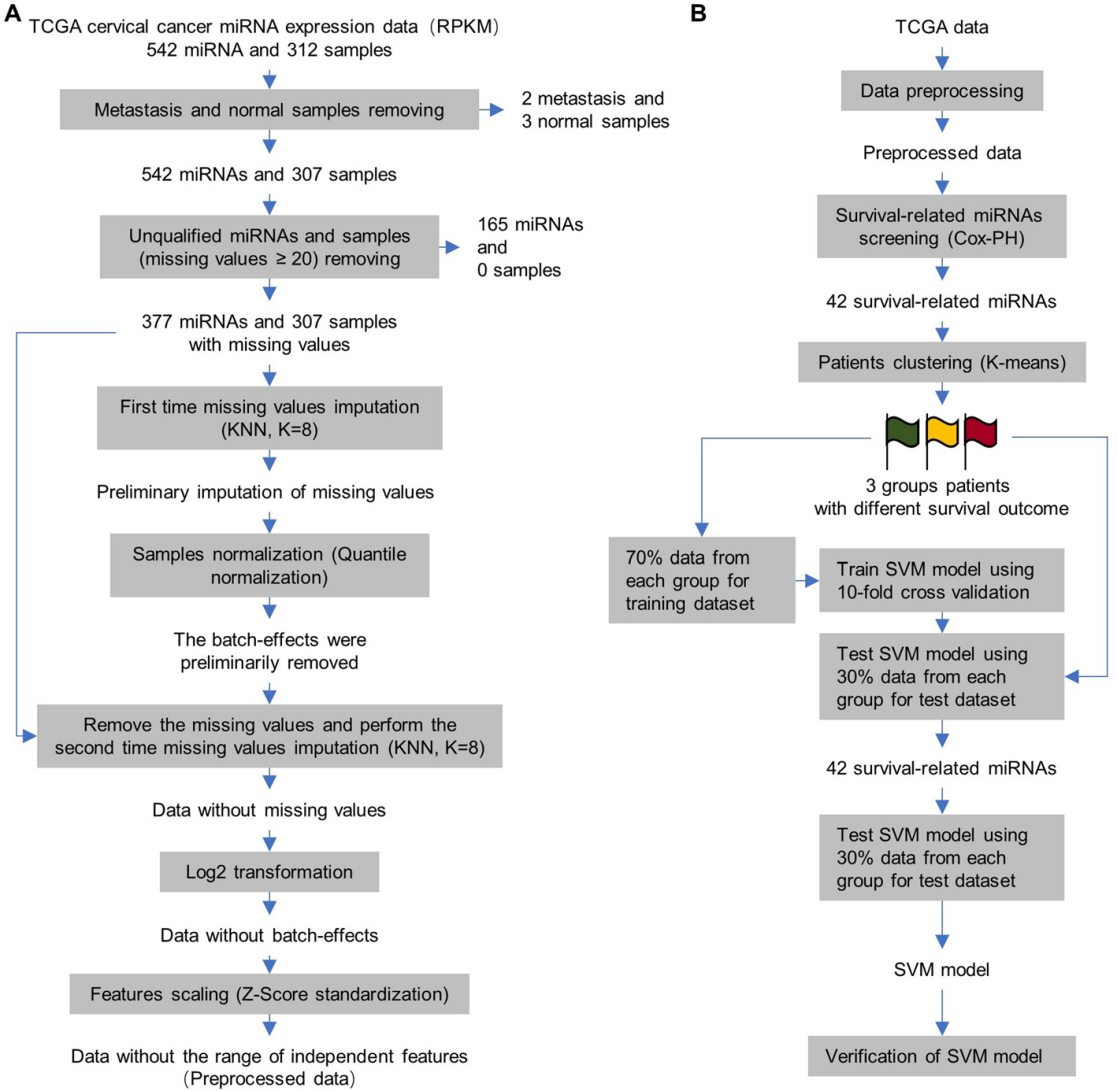
The workflow of data preprocessing and prediction model development. **a** The workflow of data preprocessing. **b** The workflow of miRNAs-based machine learning cervial cancer survival prediction model development

The TCGA cervical cancer miRNA expression data (RPKM) was downloaded, in which 542 miRNAs in 312 cervical cancer samples were included. The reduced number of miRNAs is the result of the fact that only a certain number of miRNAs express in a specific cell [25]. The workflow of data preprocessing was given in Fig. 2a. The 2 metastasis and 3 normal control samples were first removed from analysis. For missing values imputation, two independent steps were performed as both batch effects and subtypes of cancer samples derived from heterogeneity of individuals should be considered. KNN missing value imputation was performed in this study; KNN is a non-parametric classification method in which K nearest neighbors of the subject were determined by physical units [26]. If the sample size is not big enough to contain K certain subtype samples in on batch, this study for example, when the batch-effects were existed in the data, K nearest neighbors are K samples in one specific batch and when the batch-effects were eliminated, K nearest neighbors are K samples in one specific molecular subtype. Thus, we performed two independent steps for missing values imputation. We first imputed the missing values with average of K nearest neighbors in one batch by KNN imputation method for subsequent sample normalization. After sample normalization by quantile normalization algorithm (the batch-effects were removed) [27], we removed the missing values and replaced them by KNN again with average of K nearest neighbors in one subtype. The data was finally processed by log2 transformation [28] and feature scaling (Z-score algorithm) [29, 30]. The miRNAs expression profiles derived from data after preprocessing was given in Additional file 1: Fig. 1.

### Survival-related miRNAs identification

**Fig. 3:**
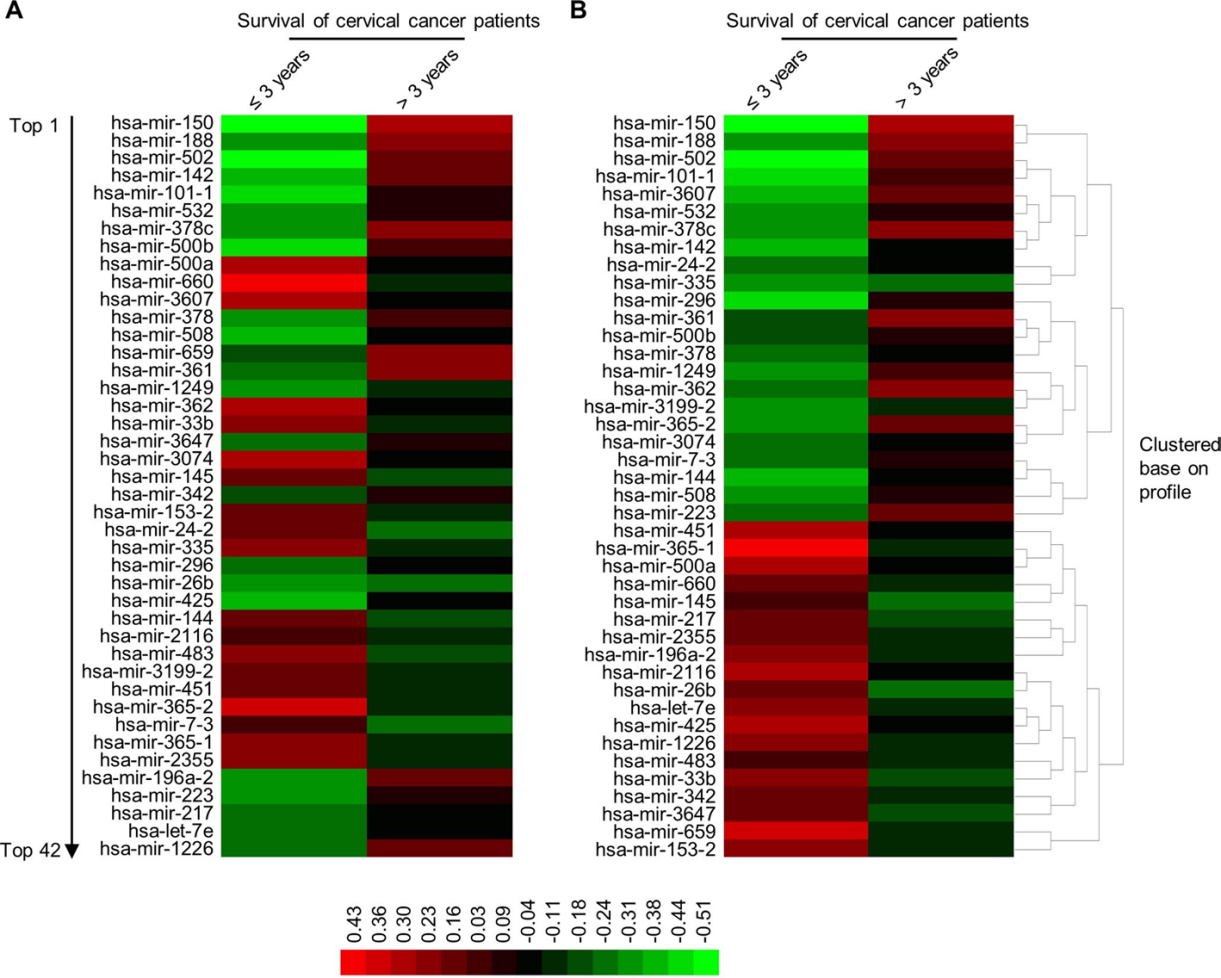
The heatmap of average expression of survival-related miRNAs in cervical cancer patients with different 3-years survival outcome. **a** The average expression level of each survival-related miRNAs identified by Cox-PH regression analysis in cervical cancer samples of patients with different 3-years survival outcome. **b** The prognosis-related miRNAs were clustered base on the expression profiles in cervical cancer samples. Cox-PH analysis was performed using coxph() function in “survival” (version 3.2–10) package and the heatmap was performed by Heml software. RStudio (version 3.6.1, RStudio, Inc.) is used

As shown in workflow of whole study (Fig. 2b), To develop the survival prediction model for cervical cancer, the features with high discriminative ability for cervical cancer survival are needed to be identified. MiRNA features were chosen as their important roles in cellular regulation and relative cost-effective for laboratory test. Cox-PH hazards model was used in this study. In total, 42 survival-related miRNAs were identified with log-rank *p* value less than 0.05; 23 and 19 miRNAs were positively and negatively correlated with survival ability of cervical cancer patients, respectively (Additional file 2: Table 1). The heatmap was given for exhibit the expression profiles of the survival-related miRNAs in tumor samples of patients (Additional file 1: Fig. 2), and the result show that the patients were clustered base on regular Euclidean distances derived from the expression profiles of the miRNAs. The Kaplan–meier survival curves were subsequentially plotted for visualization of the discriminative ability of these miRNAs (Additional file 1: Fig. 3). Finally, the average expression of survival-related miRNAs in tumor samples of patients with different 3-years survival outcome was presented in (Fig. 3). However, although the survival-related miRNAs were identified, as the different discriminative ability of the features, the combination of these features should be optimized for prediction model development through K-means clustering analysis.

### K-means clustering of cervical cancer patients with survival-related miRNAs

**Fig. 4:**
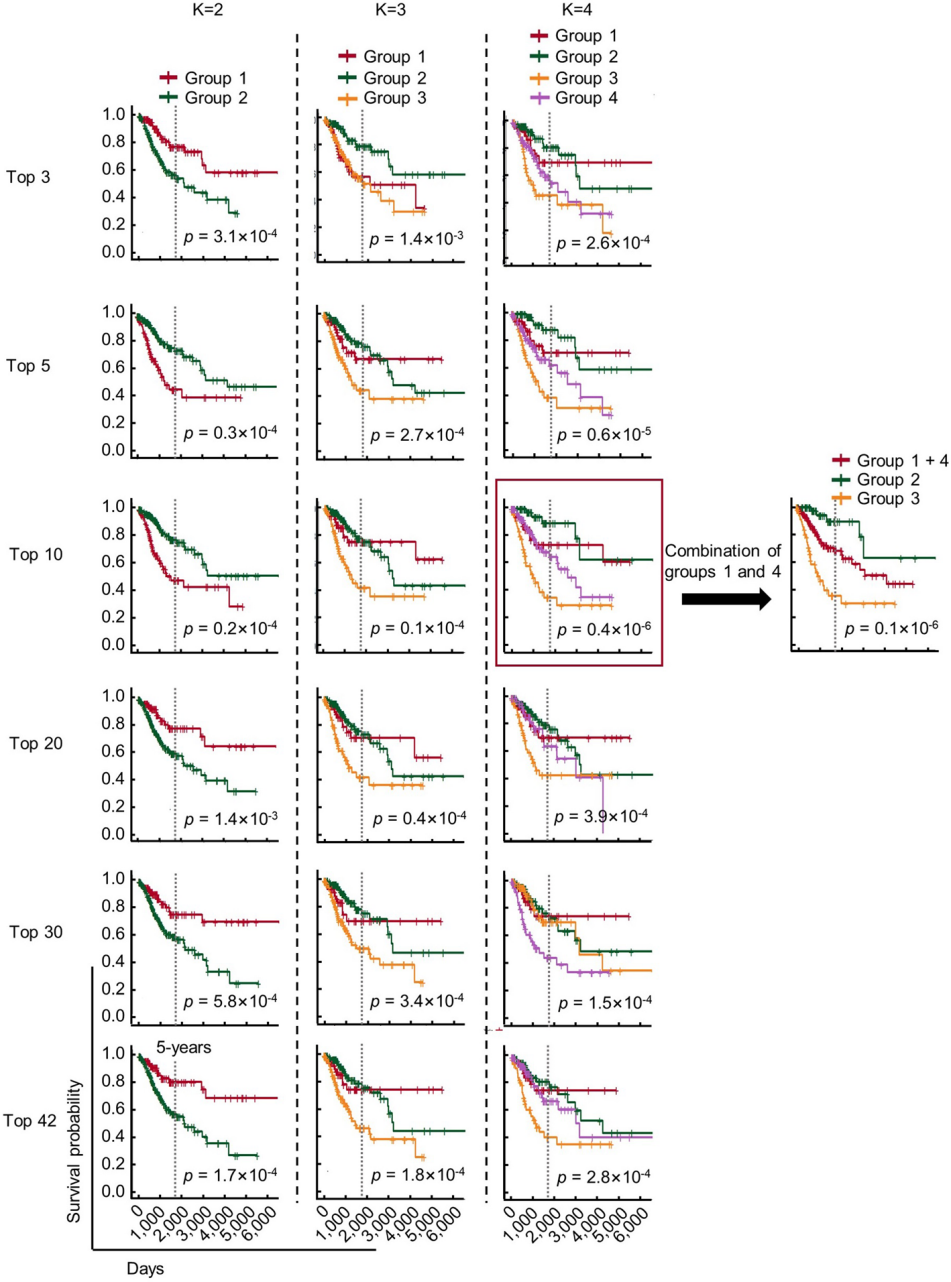
Kaplan–meier analysis of the survival of cervical cancer patients stratified by K-means clustering. The cervical cancer patients were clustered by K-means clustering algorithm (K = 2–4) with top 3, 5, 10, 20, 30, 42 survivla-related miRNAs, followed by Kaplan–meier analysis of the survival of corresponding patients. K-means clustering was performed using kmeans() in “stats” (version 3.6.2) package. Kaplan–meier analysis was performed using Surv() and survfit() function in “survival” (version 3.2–10) package. The Kaplan–meier plot was performed using ggsurvplot() function in “survminer” (version 0.4.9). RStudio (version 3.6.1, RStudio, Inc.) is used

Next, the patients were stratified by K-means clustering algorithm with survival-related miRNAs; the grouped patients will be subsequentially used for training prediction model. K-means clustering is a widely used machine learning program to partition n non-prelabeled observations into K clusters base on the characteristics of the features of the observations [31, 32]. In this study, we preformed the program when K = 2 to 4, and the number of the features were optimized. As shown in Fig. 4, Kaplan–meier curves showed that the patients with different survival outcome were successfully separated by top 3, 5, 10, 20, 30 and all 42 miRNAs when K = 2. Notably, when K = 4, the program successfully separated the patients into four groups with three obviously different survival outcome base on top 10 survival-related miRNAs features. This result indicates that, theoretically, a prediction model with the ability to stratify patients into four groups with three different survival outcome (5-years survival rate ≥ 90% (group 2), ≈ 65% (group 1 and 4) and ≤ 40% (group 3)) could be developed. Consideration of the biggest usefulness, this parameter (Top 10 miRNAs, K = 4) was used for prediction model development.

### SVM prediction model development

**Fig. 5:**
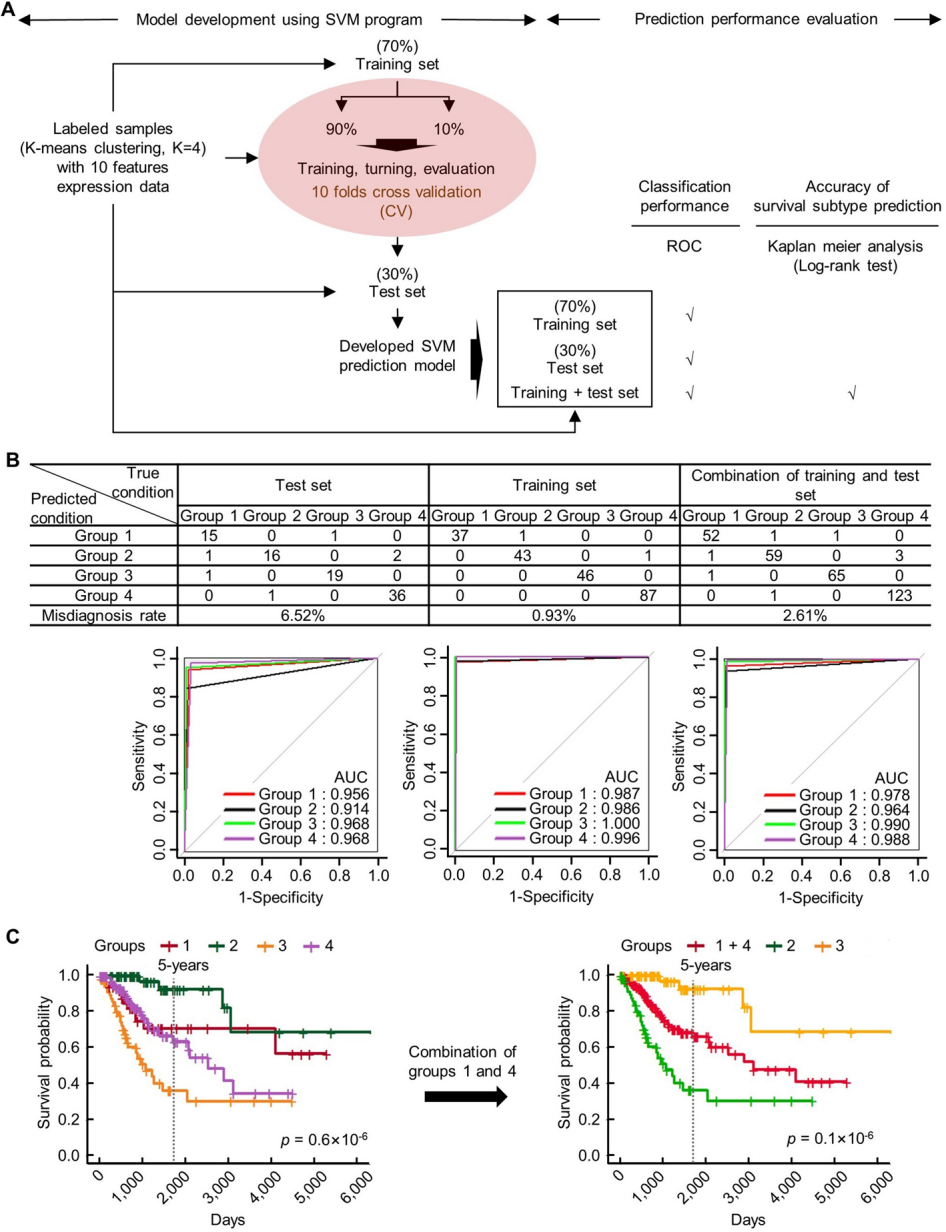
Development and evaluation of miRNAs-based machine learning cervical cancer survival prediction model. **a** Workflow of model development and evaluation. **b** The performance of classification for the prediction model was evaluated by ROC (Receiver operating characteristics) curve and AUC (Area under curve) value. **c** The accuracy of survival subtype prediction was assessed by Kaplan–meier analysis and Log-rank *p* value. SVM algorithm implementation was performed using svm() function in “e1071” (version 1.7–6) package and predict() function in “car” (version 3.0–10). ROC curve analysis was performed using roc() function in “pROC” (version 1.17.0.1) package and plot() function in “graphics” (version 3.6.1). Kaplan–meier analysis was performed using Surv() and survfit() function in “survival” (version 3.2–10) package. The Kaplan–meier plot was performed using ggsurvplot() function in “survminer” (version 0.4.9). RStudio (version 3.6.1, RStudio, Inc.) is used

Next, the K-means clustering-derived of grouped patients and the related expression data of top 10 survival-related miRNAs were subjected into SVM program for prediction model training and development. SVM is one of the most powerful prediction methods for classification- or regression-aimed data analysis base on statistical learning frameworks and Vapnik–Chervonenkis (VC) theory [33]. In this study, a 7/3 split was used for generation of training and test data and tenfold cross-validation (CV) [34] was chosen. The workflow of SVM model development and evaluation was given in Fig. 5a.

After model development, the area under the operating characteristic curve (ROC) (AUC) was used to evaluate the discriminative ability of the model. As shown in Fig. 5b, the model exhibited high performance: AUC value = 0.956 (group 1), 0.914 (group 2), 0.968 (group 3), 0.968 (group 4) for test set; 0.987 (group 1), 0.986 (group 2), 1.000 (group 3), 0.996 (group 4) for training set; 0.978 (group 1), 0.964 (group 2), 0.990 (group 3), 0.988 (group 4) for whole set, and the misdiagnosis rate was 6.52% (test set), 0.93% (training set), 2.61% (whole set), respectively. Furthermore, the groups of patients predicted by the SVM model exhibited the similar survival outcome as the patients clustered by K-means algorithm (Fig. 5c), which confirmed the accuracy of the SVM prediction model. Collectively, a miRNAs-based ML CCSPM that stratifies cervical cancer patients into high survival rate (5-years survival rate ≥ 90%), moderate survival rate (5-years survival rate ≈ 65%) and low survival rate (5-years survival rate ≤ 40%) was developed.

### Pathway analysis of the targets of miRNAs used in the model development

**Fig. 6:**
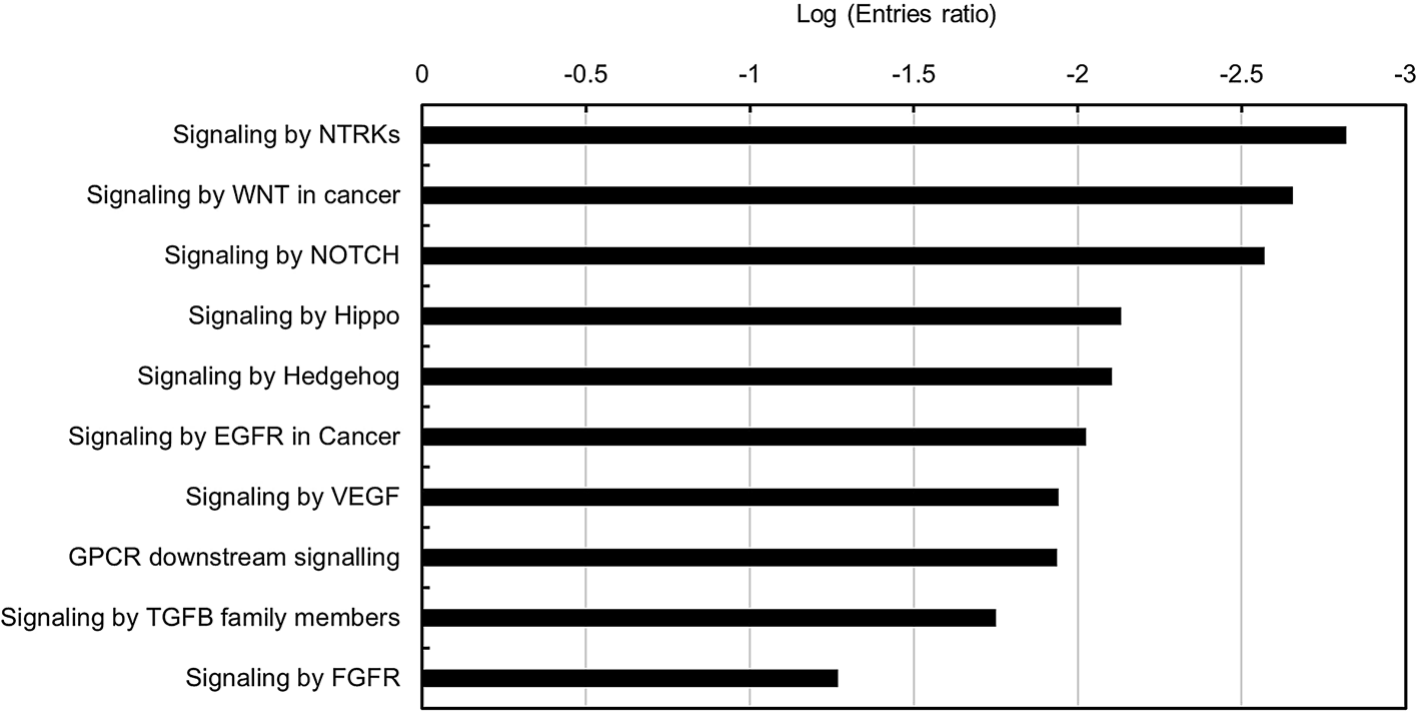
Pathway analysis of the targets of miRNAs used in model development. The mature miRNAs of survival-related stem-loop miRNAs used in model development were checked by miRbase online database (http://www.mirbase.org/). The predicted targets of mature miRNAs were analyzed by miRDB online tool (http://mirdb.org/index.html). Only top 10 targets were included for bioinformatic analysis. Pathway analysis was performed by reactome online software (https://reactome.org/)

To understand the mechanisms underlying the miRNAs that served as the features for ML CCSPM, a pathway analysis was performed with the targets of these miRNAs. As shown in Fig. 6, the targets impacted the pathways involved in cancer stem cells (CSCs). It had been recognized that CSCs are the root of cancer initiation, progression, drug resistance, that leads to treatment failure [35]. This result indicated that the mechanism underlying the miRNAs selected as the features for ML CCSPM model is their roles in CSCs regulation.

## Discussion

In this study, we developed a CCSPM base on TCGA database and machine learning technology which successfully stratified the cervical cancer patients into three groups (high survival ability, moderate survival ability, and low survival ability with 5-years survival rate ≥ 90%, ≈ 65% and ≤ 40%).

Preprocessing is important for statistical analysis of biological data; several challenges are existed in biological samples-derived big data, including missing values, batch-effects and different ranges of independent features, etc. [24]. Sample normalization and feature scaling are normally necessary for data preprocessing, which were performed for elimination of batch-effects and normalizing the range of independent feature, respectively; quantile and Z-score algorithms were well-known and wildly-used methods for these two steps [27, 29, 30]. KNN methods are most popular strategy for missing values imputation [26], while, for cancer research, both batch-effects and subtypes of cancer samples are needed to be considered; the later one is usually ignored in studies. Cancer heterogenicity refers to the existence of subpopulations of tumor cells with different genotypes and phenotypes; this heterogenicity may exist in the same tumor or tumors from different patients [36]. Thus, ideally, the missing values should be replaced by the average values derived from same subtypes of tumor samples in certain batch. However, as the high cost of omics-data extraction, the number of patients included in the database is usually not enough. Therefore, two steps missing values imputation were adopted in this study; the missing values first were imputed by KNN method with average of values in certain batch for subsequent normalization, and after normalization, the missing values were imputed by KNN method again with average of values in certain subtype of samples (Fig. 2a). The strategy employed in this study maximumly attenuate the disturbance of missing values.

Accurately forecasting the survival cancer patients are important for therapeutic decision. Currently, most molecular-based survival prediction model stratified the patients into two groups with different survival outcome [37, 38]. For example, Zhao and colleagues developed a five-genes prognostic model that stratifies the cervical cancer patients into two groups with 5-years survival rate ≈ 80% and 50%, respectively [37], while, this result is not accurate enough for therapeutic decision making. In this study, the 10 miRNAs-based prediction model developed by SVM program could robustly stratify the cervical patients into three groups (5-years survival rate ≥ 90%, ≈ 65% and ≤ 40%), which significantly improves the usefulness of the model (Table 2).

**Table 2:** Comparation of SVM model developed in this study and Zhao model

Models	Groups stratified	5-years survival rate of each group	Log-rank p value	References
This study	3	≥90%, ≈ 65% and ≤ 40%	0.1×10^-6^	
Zhao et al.	2	≈80% and 50%	<0.001	37

In this study, we used K-means algorithm to stratify patients automatically base on miRNA expression characteristics, however, medical experience of researchers is helpful for the interpretation of the meaning of the clustering result. As shown in Fig. 4, it is difficult to decide that K = 4 is the optimal clustering strategy according to the log-rank *p* value, while, although group 1 and group 4 possess distinct miRNA expression profiles, the patients show the similar 5-year survival outcome. Meanwhile, by this clustering strategy, the patients with high survival rate (group 2) and low survival rate (group 3) were stratified more accurately.

CSCs are the prime cause of cancer treatment as their congenital self-renewal capacity and enhanced metastasis, tumor-initiation and drug resistance abilities [35]. There are several pathways that play essential roles for CSCs maintenance; these pathways, such as Wnt, Hedgehog, Hippo and NOTCH etc. are also essential for normal stem cell regulation and development [39, 40]. The alteration of signaling involved in cancer microenvironment, including VEGF, FGFR, EGFR, GPCR, NTRKs and TGFB, etc. have also been found in CSCs [41–43]. Pathway analysis showed that the targets of the miRNAs used for CCSPM development significantly impacted these pathways (Fig. 6), indicating that the miRNAs associated with CSCs largely correlate with survival of cervical cancer patients and could be served as features for CCSPM development.

## Conclusion

In summary, a miRNAs-based ML CCSPM was developed that robustly stratifies cervical cancer patients into high survival rate (5-years survival rate ≥ 90%), moderate survival rate (5-years survival rate ≈ 65%) and low survival rate (5-years survival rate ≤ 40%).

## Methods

### Datasets and data analysis

The miRNA expression reads per million of mapped reads per kilobase of transcript length (RPKM) data, which includes 542 miRNAs and 312 samples, and the related clinical information were downloaded by firehose online tools (file package name: gdac.broadinstitute.org_CESC.miRseq_Preprocess.Level_3.2016012800.0.0, miRNA file name: CESC.miRseq_RPKM.txt, clinical information file name: gdac.broadinstitute.org_CESC.Merge_Clinical.Level_1.2016012800.0.0) [15]. RPKM is a method for normalization of raw counts. As a specific cell expresses a certain number of miRNAs, not all 1046 miRNAs were included in the data. All data analysis with the exception of heatmap was performed in R [44] using RStudio (version 3.6.1, RStudio, Inc.).

### Determination of the correlation between clinical features and survival of cervical cancer

The samples of metastasis tumor and normal tissues were first removed. The selected clinical features were grouped as shown in Fig. 2a and the patients in each group were divided based on 3-years survival (Fig. 2a). Chi-square test was used to determine the correlation between clinical features and 3-years survival of the patients. *P* values less than 0.05 were regarded as statistically significant. The potential features (*P* less than 0.1 by Chi-square analysis) were further confirmed by Kaplan–meier analysis. Chi-square was performed using chisq.test() function in “stats” (version 3.6.2) package [44]. Kaplan–meier analysis was performed using Surv() and survfit() function in “survival” (version 3.2–10) package [45]. The Kaplan–meier plot was performed using ggsurvplot() function in “survminer” (version 0.4.9) [46].

### Data preprocessing

The workflow of data preprocessing was presented in Fig. 3a. Unqualified samples and features removing, missing values imputation, samples normalization and features scaling were performed for miRNA expression data preprocessing. First, 2 metastasis and 3 normal samples were removed. Then, the miRNAs and samples with missing values ≥ 20 were removed. To perform sample normalization to eliminate the batch-effects, the missing values were preliminarily imputed by K-nearest neighbors (KNN) method, which impute the missing values by the average of K nearest neighbors, which were determined by Euclidean distances, in this study. KNN analysis was performed using knnImputation() function in “DMwR2” (version 0.0.2) [48].

Sample normalization was performed by quantile normalization method as described by Zhao’s publication [27]. The batch-effects were removed by this step. As not only batch-effects but also subtypes of heterogeneous tumor cells were considered for missing values imputation, the missing values were removed and replaced again by KNN method with normalized data. After log2 transformation, the feature scaling was performed by Z-score method. The preprocessed data was provided in Additional file 3: Table 2. The source code of data preprocessing was provided in GitHub (https://github.com/dingdongyan/New-CESC-2021). Quantile normalization was performed using normalize.quantiles() function in “preprocessCore” (version 1.48.0) package [48]. Calculation of average and standard deviation for Z-score analysis were performed using mean() function in “base” (version 3.6.2) package [44] and sd() function in “stats” (version 3.6.2) package [44].

### Survival-related miRNAs identification

Cox proportional hazard (Cox-PH) model was used to identification of survival-related miRNAs. Cox-PH analysis was performed using coxph() function in “survival” (version 3.2–10) package [45]. All parameters were default. *P* values less than 0.05 were regarded as statistically significant. The expression profiles of survival-related miRNAs in patients were presented as heatmap. The heatmap was performed by Heml software [49]. The source code was provided in GitHub (https://github.com/dingdongyan/New-CESC-2021).

### K-means clustering

R command, kmeans() was used to stratify the patients base on survival-related miRNAs. The expression data of top 3, 5, 10, 20, 30 and all miRNAs were input into K-means program. The parameters: centers = 2 to 4, inter.max = 10, nstart = 1, algorithm = Hartigan-Wong, trace = TURE. The source code was provided in GitHub (https://github.com/dingdongyan/New-CESC-2021). K-means clustering was performed using kmeans() in “stats” (version 3.6.2) package [44].

### Kaplan–meier analysis

Kaplan–meier analysis was used to calculate the survival rate of stratified patients and plot the survival curve. The analysis was performed by Surv() and survfit() function in “survival” (version 3.2–10) package [45] and the plot was performed by ggsurvplot() function in “survminer” (version 0.4.9) [4]. Log-rank *p* value for each analysis was given. The source code was provided in GitHub (https://github.com/dingdongyan/New-CESC-2021).

### Supervised classification model development

The supervised classification model was developed by SVM algorithm with labeled samples and the expression data of survival-related miRNAs. The SVM model was developed by splitting the samples 70%/30% to training and held-out testing data. SVM algorithm implementation was performed using svm() function in “e1071” (version 1.7–6) package [50] and predict() function in “car” (version 3.0–10) [51]. The prime parameters: type = C-classification, kernel = radial, gamma = 0.05, cross = 10, cost = 5, scale = FALSE. The source code was provided in GitHub (https://github.com/dingdongyan/New-CESC-2021).

### ROC curve analysis

ROC curve analysis was performed using roc() function in “pROC” (version 1.17.0.1) package [52] and plot() function in “graphics” (version 3.6.1) [44]. The source code was provided in GitHub (https://github.com/dingdongyan/New-CESC-2021).

### Bioinformatic analysis

The mature miRNAs of survival-related stem-loop miRNAs were checked by miRbase online database [53]. The predicted targets of mature miRNAs derived from survival-related miRNAs used for SVM model development were analyzed by miRDB online tool [54]. The top 10 targets were included for bioinformatic analysis. Pathway analysis was performed by reactome online software [55].

## Supplementary Information

**Additional file 1**

**Additional file 2**

**Additional file 3**

## Data Availability

The datasets supporting the conclusions of this article are included within the article and its additional files. The codes are deposited in GitHub (https://github.com/dingdongyan/New-CESC-2021).

## Abbreviations

HPV: Human papilloma virus
miRNA: MicroRNA
lncRNA: Long non-coding RNA
TCGA: The cancer genome atlas
ML: Machine learning
CCSPM: Cervical cancer survival prediction model
KNN: K-Nearest Neighbor
Cox-PH: Cox Proportional-Hazards
SVM: Support vector machine
ROC: Receiver operating characteristics
AUC: Area under the curve
CSCs: Cancer stem cells
