# Supplementary material for: Machine learning-based prediction of survival prognosis in cervical cancer
Source: BMC Bioinformatics. 2021 Jun 16;22:331. doi: 10.1186/s12859-021-04261-x (PMC8207793; doi:10.1186/s12859-021-04261-x)
Supplement: Supplementary file 1 — Additional file 1 [file 12859_2021_4261_MOESM1_ESM.docx]

Supplementary Materials for

**Machine learning-based prediction of survival prognosis in cervical cancer**

Dongyan Ding^1,2#^, Tingyuan Lang^1,2,3#*^, Dongling Zou^2^, Jiawei Tan^4^, Jia Chen^4^, Lei Zhou^5,6,7^, Dong Wang^2^, Rong Li^2^, Yunzhe Li^1,2^, Jingshu Liu^1,2^, Cui Ma^8^, Qi Zhou^1,2,3*^

^1^Key Laboratory of Biorheological Science and Technology (Chongqing University), Ministry of Education, Bioengineering College, Chongqing University, Chongqing, 400044, People's Republic of China; ^2^Department of Gynecologic Oncology, Chongqing University Cancer Hospital, School of Medicine, Chongqing University, Chongqing, 400030, People's Republic of China; ^3^Chongqing Key Laboratory of Translational Research for Cancer Metastasis and Individualized Treatment, Chongqing University Cancer Hospital, School of Medicine, Chongqing University, Chongqing, 400030, People's Republic of China; ^4^School of Mathematics and Statistics, Changchun University of Technology, Changchun, 130012, People's Republic of China; ^5^Singapore Eye Research Institute, The academia, 20 College Road, Discovery Tower Level 6, Singapore, 169856, Singapore; ^6^Department of Ophthalmology, Yong Loo Lin School of Medicine, National University of Singapore, Singapore; ^7^Ophthalmology and Visual Sciences Academic Clinical Research Program, Duke-NUS Medical School, National University of Singapore, Singapore; ^8^Department of Pediatric Hematology, First hospital of Jilin University, Changchun 130023, Jilin, People's Republic of China.

**# Dongyan Ding and Tingyuan Lang contributed equally to this work.**

*Correspondence: Qi Zhou, Department of Gynecologic Oncology, Chongqing University Cancer Hospital, School of Medicine, Chongqing University, Chongqing, 400030, People's Republic of China, ([cqzl_zq@163.com](mailto:cqzl_zq@163.com)) and Tingyuan Lang, Department of Gynecologic Oncology, Chongqing University Cancer Hospital, School of Medicine, Chongqing University, Chongqing, 400030, People's Republic of China, ([michaellang2009@163.com](mailto:michaellang2009@163.com)).

Supplementary Figures

Figure. S1.

**
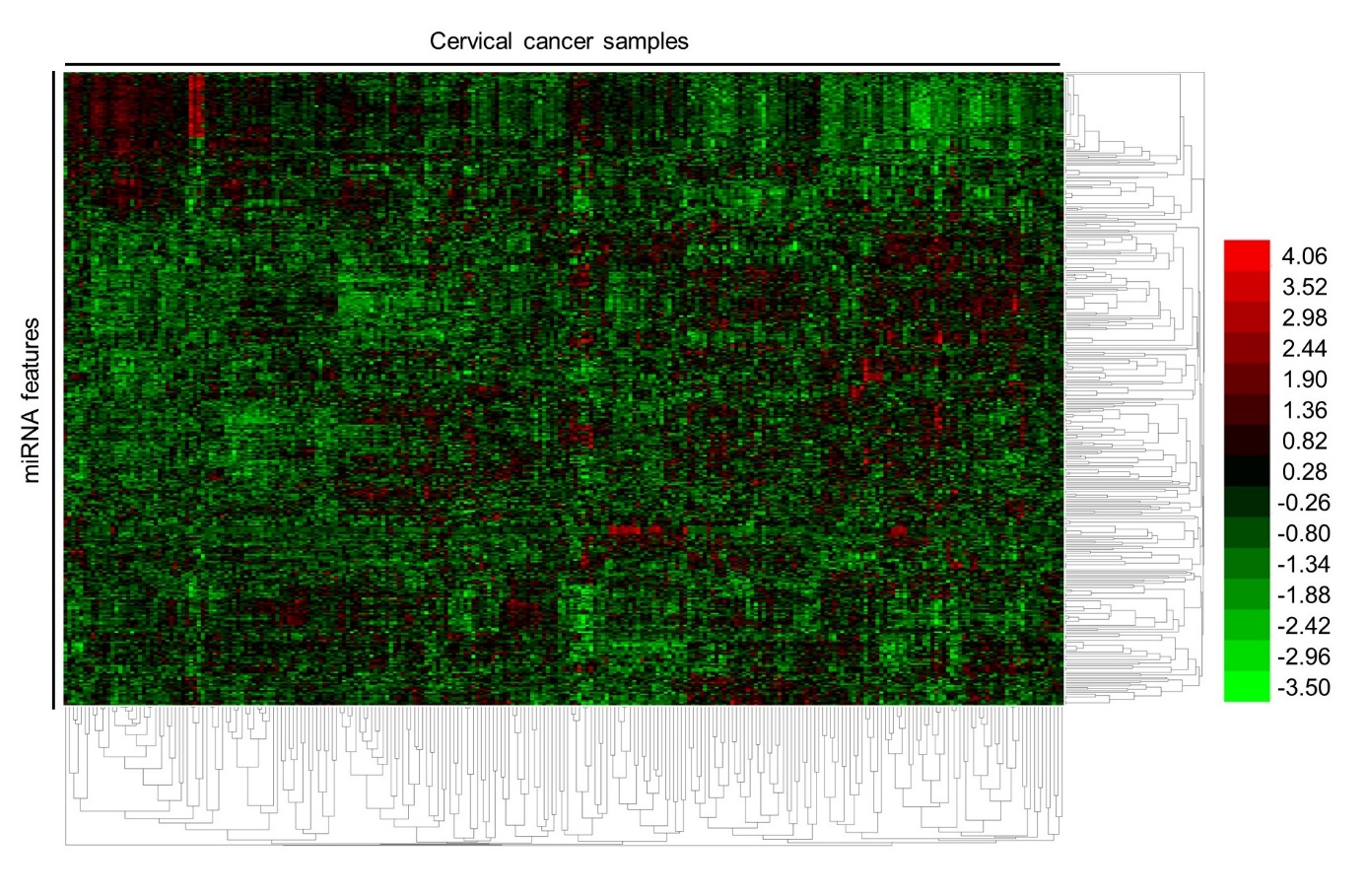
**

**Supplementary Figure S1. Expression prolife of miRNAs in cervical cancer samples.**

The miRNA expression data was downloaded in TCGA database and preprocessed. The expression profiles of miRNAs in cervical cancer samples derived by preprocessed data were exhibited as heatmap. the heatmap was performed by Heml software.

Figure. S2.


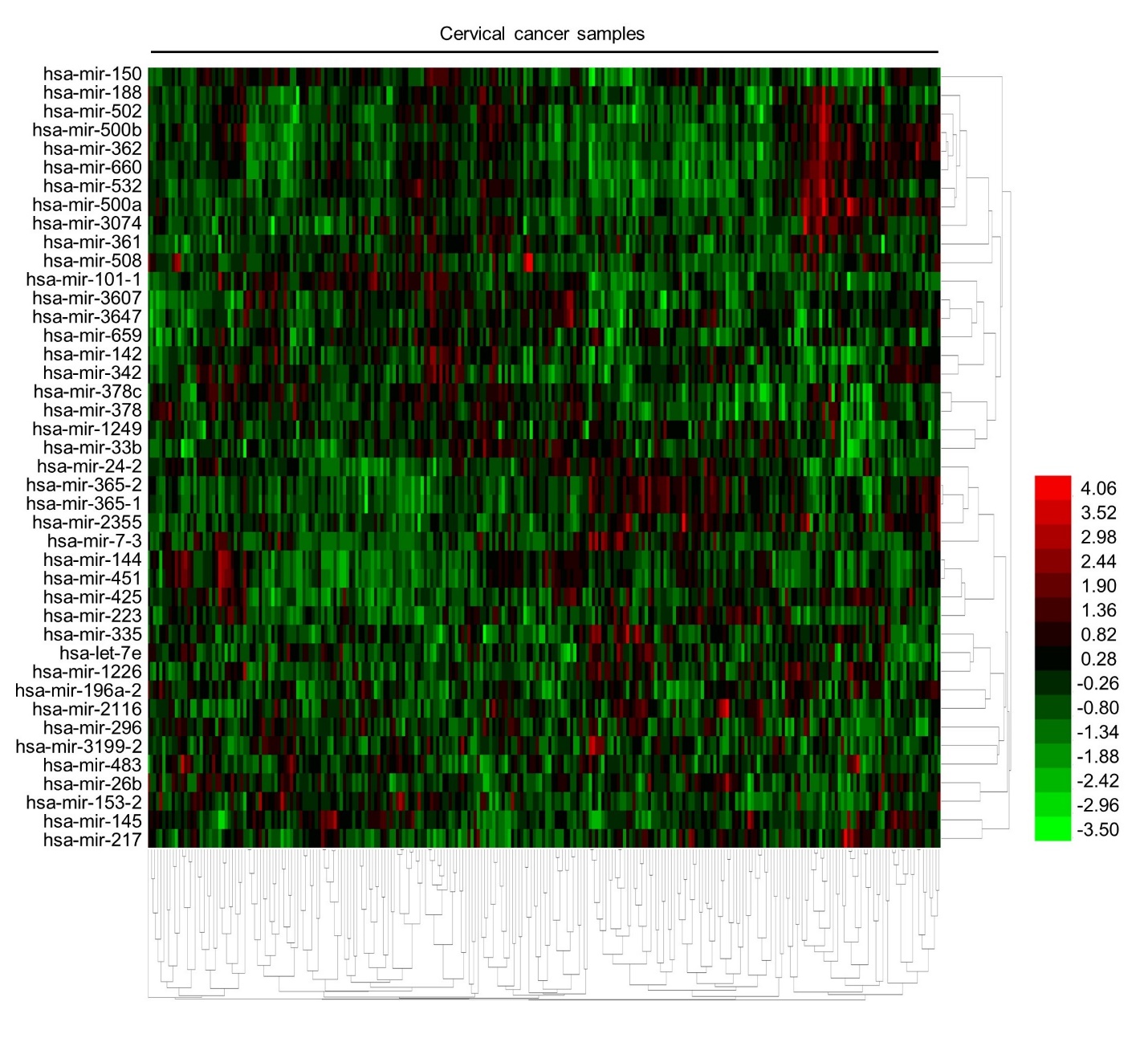


**Supplementary Figure S2. Heatmap of expression data of survival-related miRNAs identified by Cox-PH analysis.**

The survival-related miRNAs were identified by Cox-PH analysis. The expression profiles of survival-related miRNAs in cervical cancer samples were exhibited as heatmap. the heatmap was performed by Heml software.

Figure. S3.


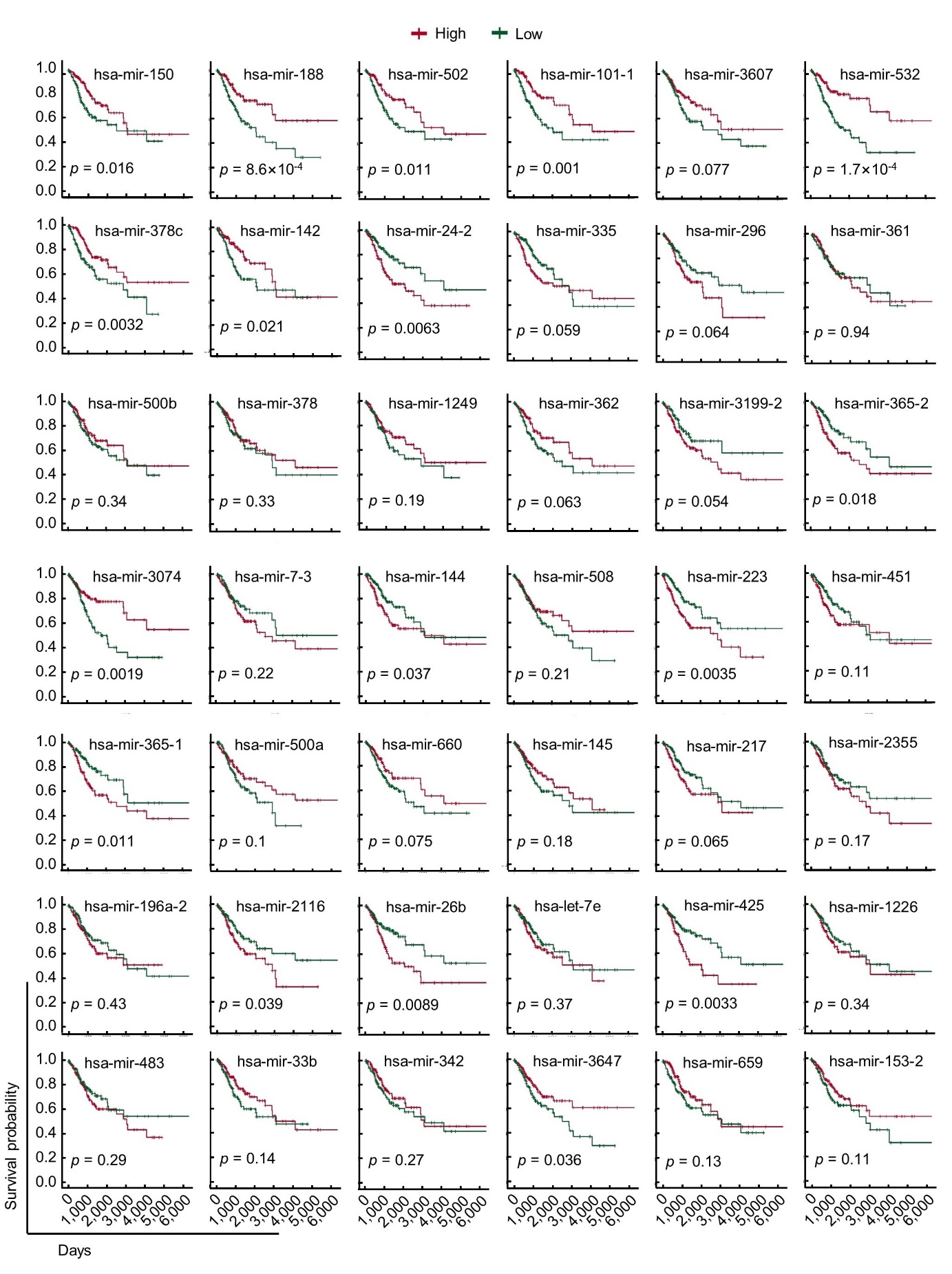


**Supplementary Figure S3. Kaplan-meier analysis of the discriminative ability of survival-related miRNAs for cervical cancer survival.**

The cervical cancer patients were stratified into two groups base on the expression level of each survival related miRNA. The Kaplan-meier analysis was performed to determine the discriminative capacity of the miRNAs. Kaplan-meier analysis was performed using Surv() and survfit() function in “survival” (version 3.2-10) package. The Kaplan-meier plot was performed using ggsurvplot() function in “survminer” (version 0.4.9). RStudio (version 3.6.1, RStudio, Inc.) is used.
